# Supplementary material for: The causal role of circulating inflammatory markers in osteoporosis: a bidirectional Mendelian randomized study
Source: Front Immunol. 2024 Jul 18;15:1412298. doi: 10.3389/fimmu.2024.1412298 (PMC11291241; doi:10.3389/fimmu.2024.1412298)
Supplement: Supplementary file 1 [file DataSheet_1.zip › supplementary material/OSTEOPOROSIS-CIM/GCST90274822.tsv.gz_forest.pdf]

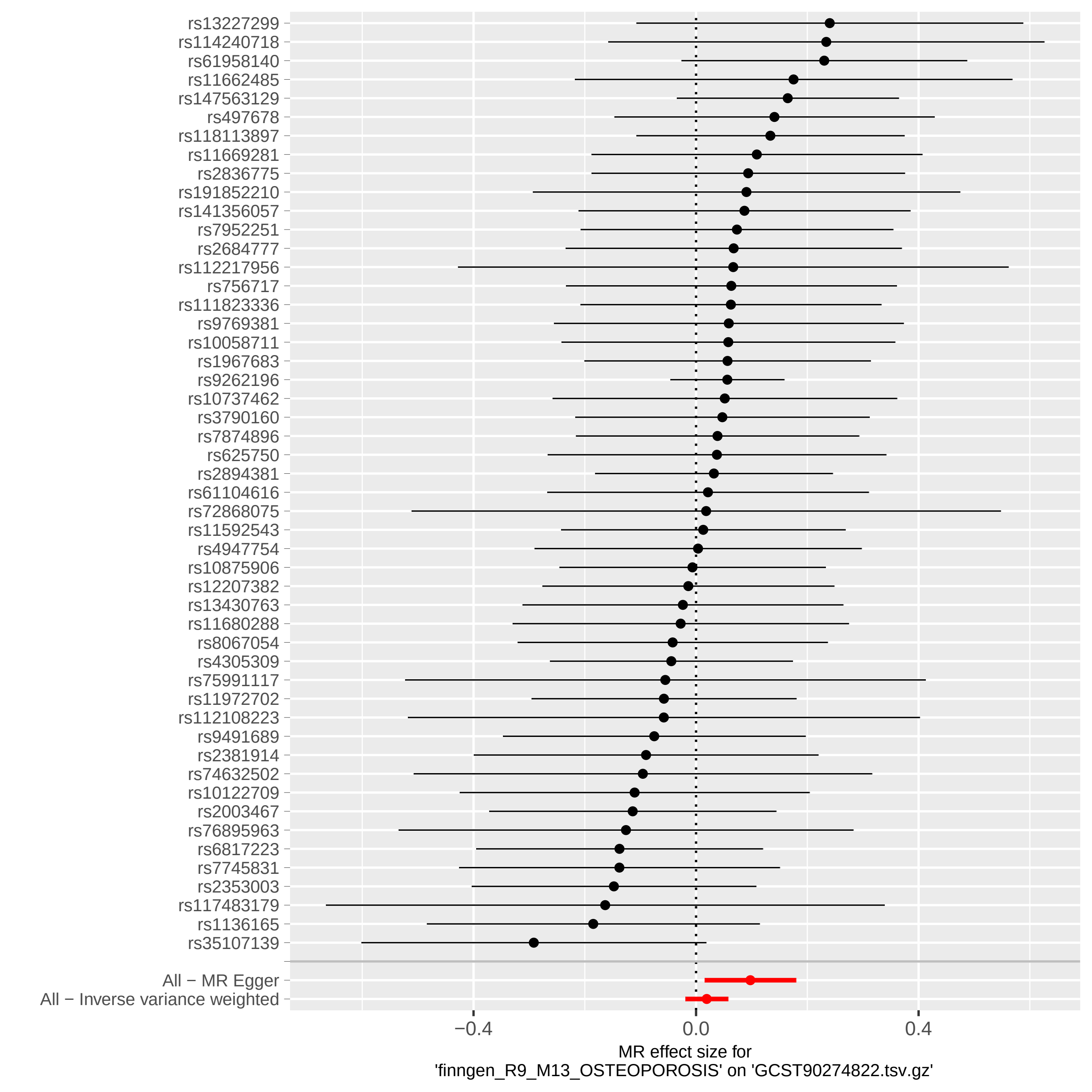

rs13227299
rs114240718
rs61958140
rs11662485
rs147563129
rs497678
rs118113897
rs11669281
rs2836775
rs191852210
rs141356057
rs7952251
rs2684777
rs112217956
rs756717
rs111823336
rs9769381
rs10058711
rs1967683
rs9262196
rs10737462
rs3790160
rs7874896
rs625750
rs2894381
rs61104616
rs72868075
rs11592543
rs4947754
rs10875906
rs12207382
rs13430763
rs11680288
rs8067054
rs4305309
rs75991117
rs11972702
rs112108223
rs9491689
rs2381914
rs74632502
rs10122709
rs2003467
rs76895963
rs6817223
rs7745831
rs2353003
rs117483179
rs1136165
rs35107139
All − MR Egger
All − Inverse variance weighted
−0.4
0.0
0.4
MR effect size for
'finngen_R9_M13_OSTEOPOROSIS' on 'GCST90274822.tsv.gz'
